# Dual-Use Vaccine for Diarrhoeal Diseases: Cross-Protective Immunogenicity of a Cold-Chain-Free, Live-Attenuated, Oral Cholera Vaccine against Enterotoxigenic *Escherichia coli* (ETEC) Challenge in BALB/c Mice

**DOI:** 10.3390/vaccines10122161

**Published:** 2022-12-16

**Authors:** Tew Hui Xian, Subramani Parasuraman, Manickam Ravichandran, Guruswamy Prabhakaran

**Affiliations:** 1 Department of Biotechnology, Faculty of Applied Sciences, AIMST University, Bedong 08100, Malaysia; 2 Department of Pharmacology, Faculty of Pharmacy, AIMST University, Bedong 08100, Malaysia; 3 Centre of Excellence for Vaccine Development (CoEVD), AIMST University, Bedong 08100, Malaysia; 4 Mygenome, ALPS Global Holding, Kuala Lumpur 50400, Malaysia

**Keywords:** live cholera vaccine, cold chain free, cholera toxin, heat-labile toxin, *Vibrio cholerae* O139, enterotoxigenic *Escherichia coli* (ETEC), combined vaccines

## Abstract

In low- and middle-income countries, diarrhoeal diseases are the second most common cause of mortality in children, mainly caused by enterotoxin-producing bacteria, such as *Shigella*, *Vibrio*, *Salmonella*, and *Escherichia coli*. Cholera and traveller’s diarrhoea are caused by *Vibrio cholerae* (O1 and O139 serogroups) and enterotoxigenic *Escherichia coli* (ETEC), respectively. The cholera toxin (CT) produced by *V. cholerae* and the heat-labile enterotoxin (LT) of ETEC are closely related by structure, function, and the immunological response to them. There is no exclusive vaccine for ETEC; however, cholera vaccines based on the CT-B component elicit a short-term cross-protection against ETEC infection. In this context, the cross-protective efficacy of MyChol^TM^, a prototype cold-chain-free, live-attenuated, oral cholera vaccine against *V. cholerae* O139 was evaluated in BALB/c mice. The 100% lethal dose (LD_100_) of 10^9^ CFU/mL of the ETEC H10407 strain was used for the challenge studies. The mice immunised with MyChol™ survived the challenge by producing anti-CT antibodies, which cross-neutralised the LT toxin with no body weight loss and no sign of diarrhoea. Compared to unimmunised mice, the immunised mice elicited the neutralising antitoxin that markedly decreased ETEC colonisation and fluid accumulation caused by ETEC H10407 in the intestines. The immunised mice recorded higher antibody titres, including anti-CT IgG, anti-LT IgG, anti-CT-B IgG, and anti-LTB IgG. Only a two-fold rise in anti-CT/CT-B/LT/LT-B IgA was recorded in serum samples from immunised mice. No bactericidal antibodies against ETEC H10407 were detected. This investigation demonstrates the safety, immunogenicity, and cross-protective efficacy of MyChol^TM^ against the ETEC H10407 challenge in BALB/c mice.

## 1. Introduction

Diarrhoeal diseases caused by bacteria, viruses, protozoa, and fungi are the second leading cause of morbidity and mortality in children under the age of five years worldwide. Among the diarrhoeal infections, *Vibrio cholerae* and enterotoxigenic *Escherichia coli* (ETEC) are enteric pathogens that cause cholera and traveller’s diarrhoea, respectively [1,2]. Cholera is a disease linked to poverty and is endemic in Africa and Southeast Asia. It is predominantly caused by *V. cholerae* serogroups O1 El Tor, and in Asian countries, mostly by O139, and the evolution of new toxigenic strains remains an important global health challenge [3,4,5]. Seven global cholera pandemics have occurred since 1817. Nearly 80 countries across the globe reported cholera in 2020, with a total of 323,320 cases [6]. It is estimated that there are 220 million cases of ETEC diarrhoea globally, with about 75 million episodes in children less than 5 years of age, resulting in between 18,700 to 42,000 deaths [7]. ETEC is often the first bacterial illness that children experience in endemic areas during their first three years of life, adversely affecting their physical and cognitive development [8,9,10]. Indeed, 10% to 40% of travellers develop ETEC diarrhoea within a few days of travelling to underdeveloped countries [11,12]. The pathogenesis of *V. cholerae* and ETEC is quite similar. Both *V. cholerae* and ETEC are also defined by their toxin secretions. ETEC causes watery diarrhoea through the actions of two toxins: the heat-stable toxin (ST) and the heat-labile toxin (LT). Its colonisation in the intestine is mediated by colonisation factor (CF) antigens and additional secondary adhesins. The LT-expressing ETEC is the predominant pathogen in 25% to 50% of all tested travellers’ diarrhoea cases [13]. The heat-labile toxin (LT) is one of the major virulence factors of ETEC [14]. The landscape of ETEC vaccines currently in development is focused on the immune responses against these virulence factors, such as LT or ST or both and CF antigens, in the form of live-attenuated vaccines, a mixture of whole-cell-killed and engineered ETEC strains to express one or more CF/CS antigens [15,16]. The cholera toxin (CT) is produced by *V. cholerae*. The CT produced by *V. cholerae* and the diarrhoeagenic heat-labile enterotoxin (LT) of ETEC are not identical but share structural similarity and both have similar type II secretion systems for their toxins [17,18,19]. 

The use of safe drinking water, improved sanitation and vaccination are the most effective approach to control diarrhoeal diseases. However, the increasing multidrug resistance of ETEC strains in both travellers and paediatric populations in low- and middle- income countries (LMICs) who are at a high risk for ETEC diarrhoea has hampered the effectiveness of antibiotic treatment [16,20]. There is no licensed vaccine against ETEC for humans [21]. Recently, the WHO reaffirmed ETEC as a priority vaccine target [22]. Currently, there is no exclusive ETEC vaccine. However, the WHO approved a killed cholera vaccine containing rCTB component, which affords short-term cross-protection against ETEC pathology [23,24]. Available vaccines in the market are safe and demand a cold chain supply (2–8 °C) to ensure their safety and potency. Cold chain logistics are difficult to execute in resource-poor countries. Hence, maintaining a cold chain for vaccination schedules results in a high cost of vaccination, which poses a great challenge. Consequently, there is an urgent need for a cost-effective, dual protection, cold-chain-free, live vaccine to manage diarrhoeal diseases in low-resource settings. In this direction, a live-attenuated cholera vaccine strain VCUSM14P, protective against *V. cholerae* O139, was developed and patented [25]. VCUSM14P was found to be non-toxigenic with increased colonisation and immunogenic properties against infection by the *V. cholerae* O139 serogroup [26]. The description of the construction of the VCUSM14P strain and its characteristics are summarized in [27]. This live vaccine strain mimics natural infection. A prototype cold-chain-free live cholera vaccine formulation with VCUSM14P was developed (Patent filed: PI 2018700106, Malaysia). The vaccine formulation (MyChol^TM^) is a liquid suspension consisting of 10^7^ CFU/mL of the live-attenuated vaccine strain (VCUSM14P), and it retains its potency at room temperature (25 °C ± 2 °C and RH 60% ± 5%) for 140 days [27,28]. MyChol™ mimics a natural infection, is non-reactogenic, immunogenic in vivo, and protects animals from a lethal wild-type *V. cholerae* O139 challenge [27,28].

The cholera vaccine consists of inactivated *V. cholerae*, and a recombinant cholera toxin B subunit (CT-B) confers short-term protection against travellers’ diarrhoea. The immunity against LT is predominantly directed against the B subunit component of LT (LT-B), which is 80% homologous with CT-B. The prototype vaccine (MyChol^TM^) also contains two copies of CT-B, and it overexpresses the B subunit of the cholera toxin, which may induce antibodies that cross-react with and neutralise the LT-B from ETEC more effectively. In addition, the prototype vaccine offers long-term protection due to its colonisation potential in the intestine [25]. Therefore, this present study was carried out to evaluate the cross-protective immunogenicity of the prototype cholera vaccine (MyChol^TM^) against ETEC in BALB/c mice to develop a dual-use vaccine for diarrhoeal diseases.

## 2. Materials and Methods

### 2.1. Animals

Healthy, adult female BALB/c mice (6–8 weeks) were obtained from the Animal Research and Service Centre, University Science Malaysia, Penang. The BALB/c mice were housed in polyacrylic cages at room temperature (20–25 °C and 60–65% relative humidity) with a 12 h light and 12 h dark cycle. The mice were provided water and food *ad libitum*. The mice were acclimatised to laboratory conditions for one week before the experimental protocol to minimise stress. This study was carried out with prior approval from AIMST University Human and Animal Ethics Committee (AUHAEC/FAS/2020/01). The study was conducted according to the Animal Research Review Panel guidelines.

### 2.2. Test Cholera Vaccine

MyChol™ is a liquid suspension that consists of 10^7^ CFU/mL of the live VCUSM14P strain. Normal saline was used as a negative control. Serial dilutions of the MyChol™ were made and plated onto Luria–Bertani agar and incubated for 16 h at 37 °C for the enumeration of the bacterial population.

### 2.3. Determination of the LD_100_ of ETEC H10407

In this study, three different doses (1 × 10⁷, 1 × 10⁸, and 1 × 10⁹ CFU/200 µL) were prepared with minor modifications [29]. The challenge bacterial ETEC H10407 (ATCC 35401) strain was purchased from ATCC. The bacterial strain was revived and grown in Luria–Bertani (LB) broth at 37 °C, 180 rpm, overnight. The overnight culture was diluted 1:10 into fresh LB medium, grown to an OD_600_ of ~1.0, and washed with sterile normal saline; then, serially diluted to desired CFU/200 µL concentration, as mentioned below, for performing the challenge study. A total of twenty-four female BALB/c were randomly divided into four experimental groups with six mice per group as detailed below.

Group I: Control group (normal saline).

Group II: H10407 (1 × 10⁷ CFU/200 µL).

Group III: H10407 (1 × 10⁸ CFU/200 µL).

Group IV: H10407 (1 × 10^9^ CFU/200 µL).

The mice were fasted for 24 h before the experiment. The mice were orally gavaged with 200 µL of the challenge strain at different doses, respectively. During the experimental period, changes in body weight, general behaviour, clinical symptoms, and the mortality of the mice were monitored and recorded. An autopsy was performed when the mice died during the experimental period, and the intestines were collected for histopathological analysis. The LD_100_ of the challenge dose determined was used as the challenge dose in MyChol™-immunised mice.

### 2.4. Cross-Protective Efficacy of MyChol™ against ETEC H10407

A total of 90 female BALB/c mice were randomly divided into three experimental groups with thirty mice per group (n = 30) as detailed below.

Group A: Control group.

Group B: Unimmunised mice challenged with H10407 (1 × 10^9^ CFU/200 µL).

Group C: MyChol^TM^-immunised mice (1 × 10^7^ CFU/200 µL) challenged with H10407 (1 × 10^9^ CFU/200 µL).

Group A animals (normal control) were administered with normal saline. Group B animals were unimmunised mice challenged with ETEC H10407 (1 × 10^9^ CFU/200 µL). Group C animals were administered with the MyChol^TM^ vaccine on day 0 and were given a booster dose on day 14. The blood samples were collected through the retro-orbital sinus on days 0, 14, 28, and 42. The serum samples were isolated for immunological analysis. Group C animals were challenged with ETEC H10407 (1 × 10^9^ CFU/200 µL) on day 28 and were monitored for next 14 days. During the experimental period, changes in body weight, general behaviour, clinical symptoms, and the mortality of the mice were monitored. If any mortality was observed, the dead animals were directly autopsied; the intestine sample was collected for fluid accumulation assay, bacterial colonisation assay, and histopathological analysis.

**Figure 1:**
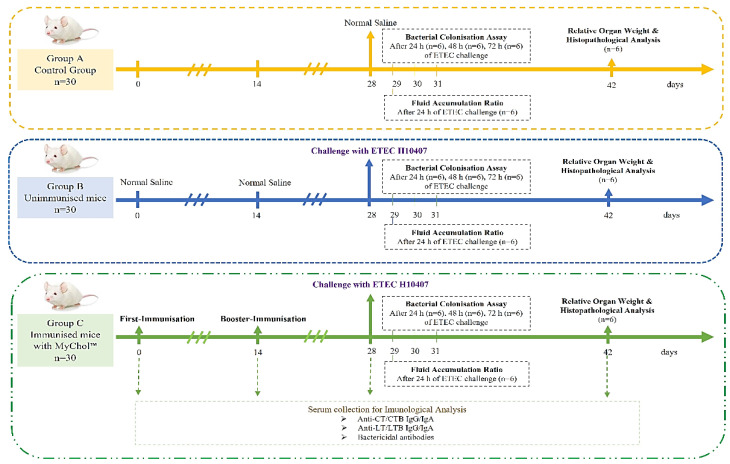
Flow diagram of evaluation of cross-protection efficacy of MyChol™ against ETEC H10407 in BALB/c mice.

After 14 days of ETEC challenge, all the animals were euthanized, and the intestine sample was collected to check the fluid accumulation ratio and bacterial colonisation. The organs, such as the brain, heart, kidney, pancreas, liver, stomach, lungs, and intestine, were collected for relative organ weight and histopathological analysis. The immunisation scheme for the evaluation of the cross-protective efficacy of MyChol™ against ETEC H10407 is depicted in Figure 1.

#### 2.4.1. Mice Immunisation

Prior to the immunisation, mice (Group C) were fasted for 24 h. They were orally gavaged with MyChol™ (1 × 10^7^ CFU/200 µL) for first immunisation on day 0 and a booster dose given on day 14. Normal saline was used as a negative control for Group B unimmunised mice. Blood samples were collected from pre and post-immunised mice on days 0, 14, 28, and 42 by sinus orbital, as previously described [28,30]. The blood samples were kept at room temperature for 2 h to allow clotting, followed by centrifugation (3500 rpm, 15 min at 4 °C) to separate the serum. The serum samples were then stored at −20 °C until use. The specificity of antibody response against CT and LT, such as anti-CT/CT-B IgG, anti-CT/CT-B IgA, anti-LT/LT-B IgG, and anti-LT/LT-B IgA, in mice was determined by ELISA assay [29]. Throughout the study period, changes in body weight, general behaviour, clinical symptoms, and the mortality of the mice were monitored and recorded. 

#### 2.4.2. ETEC Challenge Study

The ETEC challenge study on unimmunised (Group B) and immunised mice (Group C) was performed to ascertain the cross-protective efficacy of MyChol^TM^ against ETEC H10407. Group A was control group without any treatment. The Group B and Group C mice were orally challenged with a lethal dose of ETEC strain H10407 (1 × 10^9^ CFU/200 µL) on day 28. Animals were closely observed up to 14 days to record their mortality percentage, the loss of body weight, and clinical signs. At the end of the study, the mice were sacrificed under mild diethyl ether anaesthesia, and the intestines were collected for fluid accumulation ratio and bacterial colonisation assay. The other organs, such as the brain, heart, kidney, pancreas, liver, stomach, and lungs, were collected for relative organ weight and histopathological analysis.

#### 2.4.3. Immunological Analysis

##### Detection of Anti-Cholera Toxin (Anti-CT), Anti-Heat Labile (Anti-LT) and Anti-Subunit Antibodies

Anti-CT or -B and anti-LT or -B serum antibody ELISAs were performed on mice serum samples with wells coated with 0.5 μg of antigen and quantified with an external mouse IgG or IgA standard, as previously described [28]. Briefly, the ELISA plates (MaxiSorp, Nunc, Roskilde, Denmark) were coated with 0.5 μg/well of antigen (CT/LT/CT-B/LT-B) in 60 mM carbonate buffer (pH 9.6) and incubated at 4 °C for 16 h. The plates were then blocked with 5% skim milk and incubated at 37 °C for 1 h. The wells were washed 3 times with wash buffer (PBS—Tween 20), and 100 μL of each sera sample (1:10–1:1280 diluted in PBS) was added and incubated at 37 °C for 2 h. The anti-cholera toxin, as a primary antibody, was used as the positive control, and PBS without any serum was used as the negative control. The plates were washed again with wash buffer, and 100 μL of anti-mouse IgG conjugated with HRP (Dilution 1:5000 in PBS) was added and incubated at 37 °C for 30 min. Subsequently, the wells were washed, and 2,20-azinobis (3-ethylbenzothiazoline-6-sulphonic acid (ABTS) was added as the substrate and incubated at 37 °C in the dark for 30 min. The absorbance reading was measured at 405 nm using 495 nm as the reference wavelength in a microtitre plate reader. For the determination of anti-CT/LT/CT-B/LT-B IgA, the protocol was the same as described above, except the primary anti-CT antibodies were captured with anti-mouse-IgA-HRP diluted 1:3000 in PBS.

##### Bactericidal Assay against H10407

The immune response in the mice that were immunised with MyChol™ was evaluated by measuring bactericidal antibodies, as previously described, with minor modifications [28]. The serum samples were heated (at 56 °C for 30 min) to inactivate the complement. A series of two-fold dilutions of serum samples in PBS was made (1:10 to 1:1280). The 25 μL of diluted serum samples were added to each well in a 96-well microtitre plate. The overnight grown ETEC H10407 strain in LB broth at 37 °C was diluted with PBS containing 20% complement to a final concentration of 10^3^–10^5^ CFU/mL. This cell suspension (25 μL) was added to each well in the microtitre plate and incubated for 60 min. After 60 min, 150 μL of pre-warmed LB broth was added to each well and incubated for 4 h. The optical densities were measured at 600 nm with a microtitre plate reader. The bactericidal antibody titre was defined as the highest serum dilution causing 100% killing of cells compared to the pre-immune sera.

#### 2.4.4. Observations

##### Body Weight Analysis and Clinical Signs

The body weights of the mice were monitored at regular intervals. Clinical signs, such as piloerection, prostration, involuntary movements, ataxia, excitation or depression, diarrhoea, incoordination, and salivation were observed daily.

##### Fluid Accumulation Ratio (FAR) in Mice after ETEC Challenge

The fluid accumulation in the intestine was collected 24 h after the ETEC challenge studies from experimental mice Group A (control group), Group B (unimmunised mice after ETEC challenge), and Group C (immunised mice after ETEC challenge). The fluid accumulation ratio (FAR) was obtained by dividing the volume of fluid (mL) accumulated per intestine by the length (cm). A ratio of equal to or greater than 1.0 was used to indicate an adverse response, whereas a ratio of equal to or less than 0.2 was used to identify a negative response [28].

##### Bacterial Colonization

Studies of the bacterial colonisation in different groups were evaluated, as previously described, with minor modifications [31]. Six mice from each experimental group: Group A (control group), Group B (unimmunised mice after ETEC challenge), and Group C (immunised mice after ETEC challenge) were sacrificed at each timepoint (24, 48, and 72 h) after lethal ETEC challenge to check for bacterial colonisation. The small intestines were collected and were homogenized in 5 mL of PBS, and serial dilutions were plated onto LB agar for CFU counting.

##### Relative Organ Weight Analyses

At the end of the study, the mice in all the experimental groups were sacrificed, organs such as the brain, heart, liver, kidneys, lungs, spleen, stomach, and intestines were excised, and relative organ weight was recorded. The relative organ weights were calculated based on the organ-to-body weight ratios.

##### Histopathological Analysis

Part of the brain, heart, lung, liver, kidney, spleen, pancreas, and intestine tissues were preserved in 10% neutral formalin for histopathological analysis. The tissue samples were embedded in paraffin after being dehydrated in alcohol and subsequently cleared with xylene. Five-micrometre-thick samples of the liver and kidney sections were prepared from the paraffin blocks, stained with haematoxylin and eosin, and mounted in a neutral DPX medium; then, the sections were examined under a light microscope.

### 2.5. Statistical Analysis

The mean ± standard error of the mean (SEM) values was calculated for each group. All data were analysed using one-way ANOVA followed by Tukey’s post hoc test. *p* < 0.001 was considered statistically significant.

## 3. Results

### 3.1. Determination of the LD_100_ of ETEC H10407

**Figure 2:**
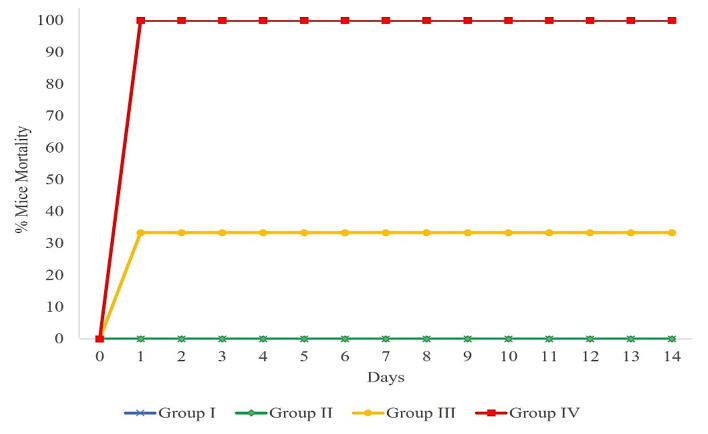
Percentage of mice mortality after challenge with ETEC H10407 strain for 14 days. Group I: normal saline (control group), n = 6; Group II: H10407 (10⁷ CFU), n = 6; Group III: H10407 (10⁸ CFU), n = 6; and Group IV: H10407 (10⁹ CFU), n = 6.

Three different challenge doses (1 × 10^7^, 1 × 10^8^, 1 × 10^9^ CFU/200 µL) of ETEC H10407 were administered in mice, and their mortality percentages are shown in Figure 2. A 100% mortality rate was observed within 24 h in Group IV mice that were infected with a dose of 1 × 10^9^ CFU/200 µL. Whereas Group III mice, infected with a dose of 1 × 10^8^ CFU/200 µL, showed a 33.33% mortality rate within 24 h. An autopsy was performed on dead mice, and the intestine appeared with more fluid accumulation in the small intestine. However, no mortality was observed in Group II mice infected with 1 × 10^7^ CFU/200 µL of H10407. The results demonstrate that 1 × 10^9^ CFU/200 µL of ETEC H10407 was found to be a 100% lethal dose (LD_100_) in BALB/c mice. In the surviving mice in Group II and III, no body weight loss was observed, and none of the mice appeared ill or developed diarrhoea before euthanasia for 14 days.

### 3.2. Cross-Protection against ETEC H10407 in Immunised Mice

**Figure 3:**
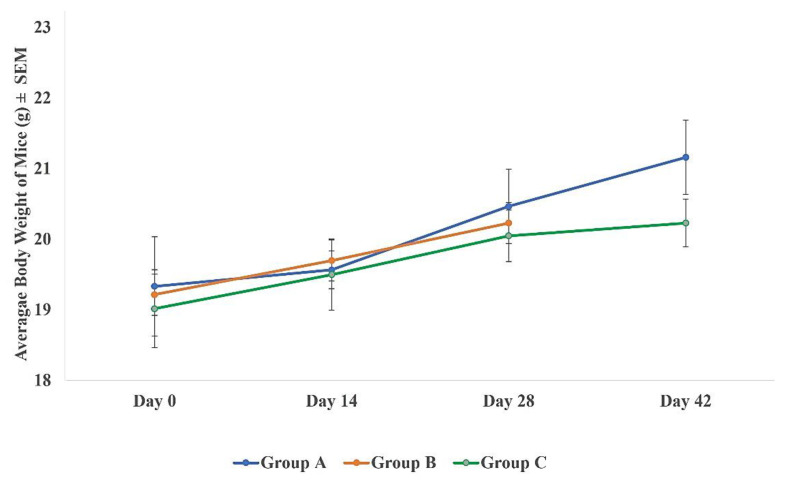
Average body weight of mice. Group A (control group, n = 6), Group B (unimmunised mice after ETEC challenge, n = 6), and Group C (immunised mice after ETEC challenge, n = 6). All the values are mean ± SEM. No significant difference in body weight was observed between Group A and Group C on day 0, 14, 28, and 42. All the mice in Group B died within 24 h after ETEC challenge on day 29; thus, no data were collected on day 42 in this experiment.

To evaluate the cross-protection efficacy of MyChol™ against ETEC, the unimmunised (Group B) and immunised (Group C) BALB/c mice were challenged with an LD_100_ dose of 10^9^ CFU/200 µL of ETEC H10407 and observed for 14 days. Group A mice were as control group in this study. The Group B mice died within 24 h after ETEC challenge. Whereas the Group C immunized mice showed a 100% survival rate (6/6) against H10407 challenge. No body weight loss (Figure 3) and clinical symptoms or mortality were observed in Group C immunised mice during the immunisation period and after challenge studies compared to Group B. Further, no abnormality and less fluid accumulation were observed in the intestine after the ETEC challenge studies.

### 3.3. Immunological Analysis

**Figure 4:**
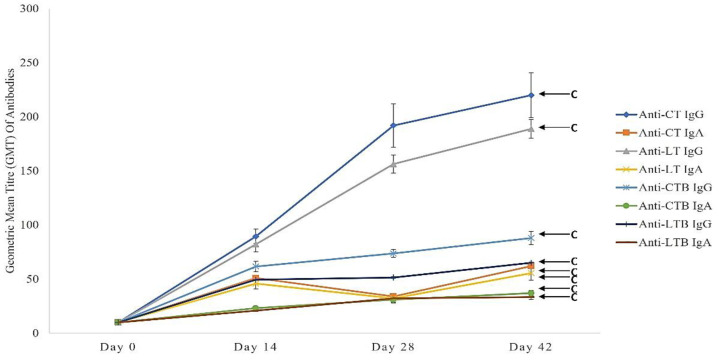
Geometric mean titre (GMT) of anti-CT/CT-B/LT/LT-B IgG, IgA antibodies elicited in immunised mice. All the values are mean ± SEM; n = 6. Significant increase (c *p* < 0.001) was observed in anti-CT/CT-B/LT/LT-B IgG, IgA antibodies on day 14, 28, and 42 compared with that of day 0. (One-way ANOVA followed by Tukey post hoc test).

The immune response of immunised mice with MyChol™ (10^7^ CFU/200 µL) was determined by measuring the anti-CT IgG, anti-CT IgA, anti-LT IgG, anti-LT IgA, anti-CT-B IgG, anti-CT-B IgA, anti-LTB IgG, anti-LTB IgA, and bactericidal antibodies. The enzyme-linked immunosorbent assay (ELISA) results show an increase in serum IgG titres to CT (18-fold), CT-B (6-fold), LT (14-fold), and LT-B (4-fold) after the booster dose (Figure 4). A two-fold increase over the baseline was observed in serum IgA titres to CT, CT-B, LT, and LT-B after the booster dose. After challenge with ETEC H10407, there was an increase in anti-CT IgG (21-fold), anti-CT IgA (5-fold), anti-LT IgG (17-fold), anti-LT IgA (4-fold), anti-CT-B IgG (7-fold), anti-CT-B IgA (3-fold), anti-LT-B IgG (5-fold), and anti-LT-B IgA (2-fold). However, no bactericidal antibodies against H10407 were detected in all the immunised mice serum samples.

### 3.4. Fluid Accumulation Ratio in Mice after ETEC Challenge

**Figure 5:**
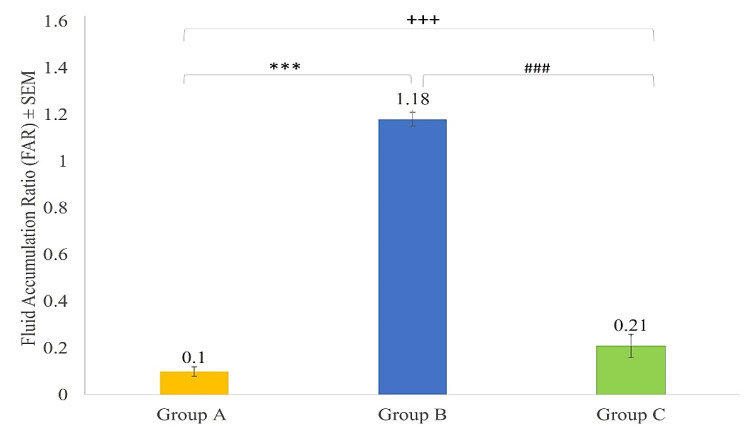
Fluid accumulation ratio (FAR) in the intestine of Group A (control group), Group B (unimmunised mice), and Group C (immunised mice) 24 h after the ETEC H10407 challenge. All the values are mean ± SEM (n = 6). *** = *p* < 0.001 compared to Group A. ### = *p* < 0.001 compared to Group B. +++ = *p* > 0.001 compared to Group C. (One-way ANOVA followed by Tukey post hoc test).

In this study, Group A (the control group) and Group C (immunised mice after ETEC challenge) both had a fluid accumulation ratio (FAR) of less than 0.2 (Figure 5). In contrast, more than 1.0 FAR was recorded in Group B (unimmunised mice after ETEC challenge) showed significant increases compared to the Group A control group (*p* < 0.001). Whereas Group C showed lower FAR when compared to Group B (*p* < 0.001).

### 3.5. Bacterial Colonisation

**Figure 6:**
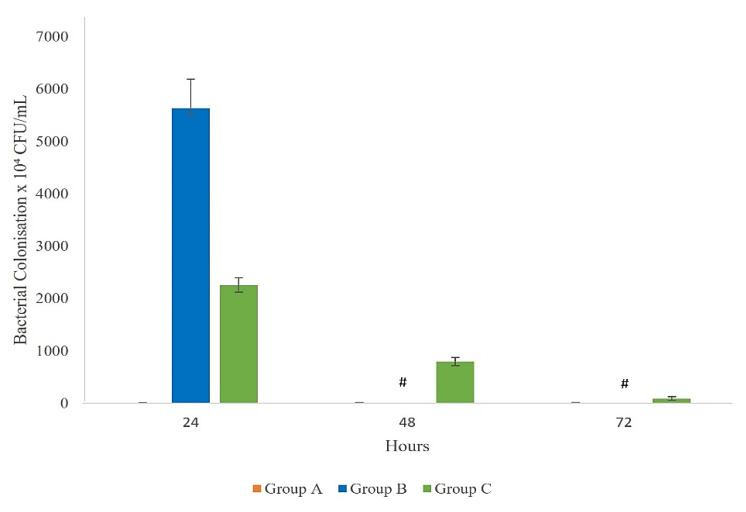
Bacterial colonisation in mice Group A (control group), Group B (unimmunised mice after ETEC challenge), and Group C (immunised mice after ETEC challenge) at 24, 48, and 72 h after lethal ETEC challenge. All the values are mean ± SEM. # = all the mice in Group B died within 24 h; thus, no data were collected at 48 and 72 h in this experiment.

The colonisation of the ETEC H10407 strain in the small intestine is critical for causing diarrhoeal disease in mice. We compared the colonization potential of the ETEC H10407 strain in immunised and unimmunised mice after a lethal ETEC challenge (Figure 6). Group A is the control group without inoculation with the ETEC H10407 strain, which recorded 10⁵ CFU/mL of microflora in the intestine. Group B unimmunised mice recorded the highest bacterial number (5.6 × 10⁷ CFU/mL) recovered from the small intestine within 24 h. No data were collected after 48 and 72 h for Group B unimmunised mice, as all the mice died within 24 h after the ETEC challenge. However, group C immunised mice recorded a lower number of bacteria (2.26 × 10⁷ CFU/mL) compared to Group B mice, and the number reduced to 8.0 × 10⁶ CFU/mL at 48 h and 9 × 10⁵ CFU/mL at 72 h.

### 3.6. Relative Organ Weights

No significant difference in the relative organ weights of the brain, heart, liver, kidneys, lungs, spleen, stomach, and intestines was observed among the experimental groups: Group A (control group), Group B (unimmunised mice after ETEC challenge), and Group C (immunised mice after ETEC challenge).

### 3.7. Histopathological Analysis

**Figure 7:**
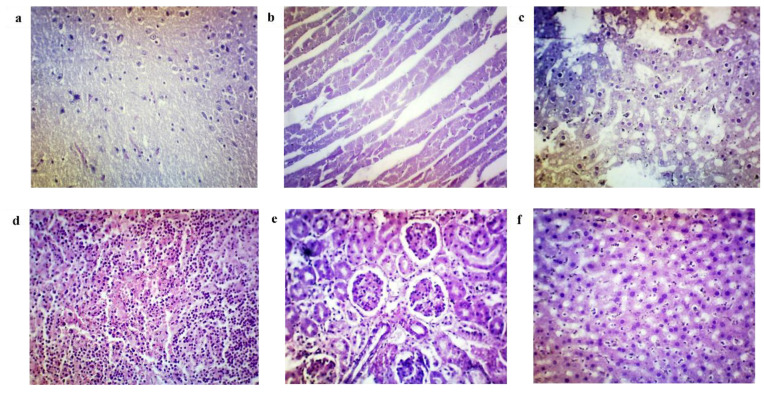
Histopathology changes in the brain (**a**), heart (**b**), lung (**c**), spleen (**d**), kidney (**e**), and liver (**f**) of unimmunised mice in Group B after ETEC challenge (H and E, ×400). (**a**) Section from the brain shows gliosis and necrotic areas. (**b**) Section from the heart shows mild degeneration of myocytes with mild interstitial oedema. (**c**) Section from a lung shows extensive lymphocytic infiltration, congestion, and oedema of the interstitium with focal alveoli oedema and type II pneumocytes hyperplasia. (**d**) Section from the spleen shows moderate focal parenchymal congestion with enlarged germinal centres, focal white pulp disintegration and apoptosis. (**e**) Section from a kidney shows mild degeneration of tubules, congestion, and moderate inflammation of the interstitium. (**f**) Section from the liver shows mild degeneration of hepatocytes and Kupfer cell hyperplasia, moderate portal inflammation and bile duct hyperplasia, and severe sinusoidal congestion.

**Figure 8:**
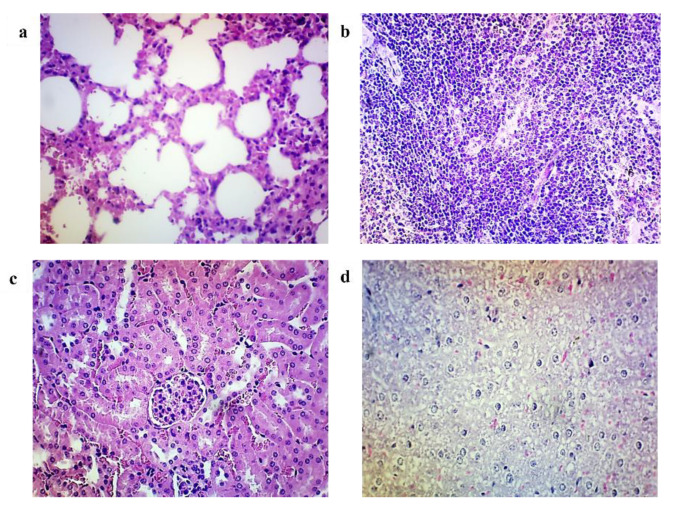
Histopathology changes in the lung (**a**), spleen (**b**), kidney (**c**), and liver (**d**) of immunised mice in Group C after ETEC challenge (H and E, ×400). (**a**) Section from a lung shows mild lymphocytic infiltration, congestion, and oedema of the interstitium with focal alveoli oedema and type II pneumocytes hyperplasia. (**b**) Section from the spleen shows mild focal parenchymal congestion and focal apoptosis. (**c**) Section from a kidney shows mild degeneration of tubules, congestion, and inflammation of the interstitium. (**d**) Section from the liver shows mild degeneration of hepatocytes, sinusoidal congestion, Kupfer cell hyperplasia, and portal inflammation.

**Figure 9:**
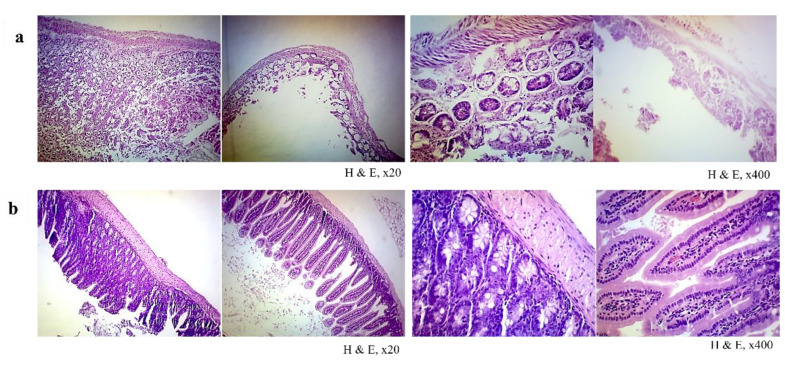
Histopathology of the intestines of unimmunised mice in Group B (**a**) and immunised mice in Group C (**b**) after ETEC challenge (H and E, ×20 and ×400). (**a**) Section from the intestines of unimmunised mice in Group B shows severe pathological alterations in the intestine, including necrosis, congestion and haemorrhage, and neutrophil infiltration. Whereas (**b**) section from the intestines of immunised mice in Group C can be seen within normal limits.

The organs from the control group (Group A) showed typical histopathological structures. The unimmunised mice after the ETEC challenge (Group B) showed a mild to severe degeneration of cells, oedema, and inflammation in most of the organ’s sections (Figure 7a–f). Whereas. the immunised mice in Group C showed mild lymphocytic infiltration in the lungs (Figure 8a), mild focal parenchymal congestion in the spleen (Figure 8b), mild degeneration of the kidney tubules (Figure 8c) and mild degeneration of liver hepatocytes (Figure 8d). After a lethal challenge with ETEC H10407, the most remarkable alterations, such as necrosis, congestion and haemorrhage, and neutrophil infiltration, were observed in Group B mice intestines (Figure 9a). It was evident that ETEC H10407 infected the intestines, and the villous epithelial cells underwent exfoliation and necrosis, congestion accompanied by haemorrhage, and neutrophil infiltration. The villi are essential parts of the small intestine, and this result proves that the lesions caused by H10407 infections are very severe. However, no abnormality and loss of microvilli were observed in the intestines of Group C immunised mice after the ETEC challenge (Figure 9b) compared to the control group, which showed the cross-protection efficacy of MyChol™. The histopathological findings of mice after ETEC challenge studies are summarized in Table 1.

## 4. Discussion

ETEC is the major cause of diarrhoeal illness in LMIC among children under five years old and a leading cause of travellers’ diarrhoea. By colonising the small intestine and secreting heat-labile toxin (LT) and heat-stable toxin (ST), ETEC induces watery diarrhoea. Both toxins attach to the intestinal epithelial cells’ surfaces, where they are then taken up by the cells and produce the cyclic nucleotides cAMP and cGMP. As a result of the activation of intracellular protein kinases by both cyclic nucleotides, ion channels are phosphorylated and altered, which leads to the buildup of salt and water in the intestinal lumen and watery diarrhoea [32,33,34]. To establish an effective infection, 10^6^–10^8^ cells of ETEC H10407 were recommended [16,35,36]. The BALB/c mouse is among the most widely used inbred animal models used in biomedical research and is particularly utilized in immunology and infectious disease research [37]. Most researchers have used BALB/c mice for the development of an ETEC vaccine and challenge studies, as BALB/c mice are susceptible to ETEC infection [38,39,40,41,42]. In mice, a lethal challenge dose of 10^8^ CFU/mL of ETEC H10407 [38,39,40] or 10^9^ CFU/mL [41,42] were reported. 

The ETEC strains commonly used in challenge studies are H10407, B7A, and E24377A [43]. In comparison, the H10407 expresses both LT and ST toxins and also colonisation factor I CFA/I and has long been recognized as the ideal strain because it causes more severe diarrhoea associated with concurrent signs and symptoms [44,45]. To test our prototype cholera vaccine’s cross-protective immunity against an ETEC challenge in BALB/c mice, we used the ETEC strain H10407 as the challenge strain. In our investigation, 10^9^ CFU of ETEC H10407 resulted in 100% mortality in the unimmunised mice within 24 h. Similar observations were reported [41,42]. In contrast, a lower dose of 10^8^ CFU also resulted in 100% mortality in mice [38,39,40]. On the other hand, in this study, 10^8^ CFU caused only 33.33% mortality. In the intestine of the dead mice, obvious fluid accumulation was observed. These observations are comparable to those obtained by [40,42,46] in mice with H10407 infections. The LT and ST toxins would have increased cAMP and cGMP levels resulting in fluid accumulation [42,46,47,48] and are similar to *V. cholerae* infections [32].

In our investigation, the MyChol^TM^-immunised mice showed good tolerability with no adverse reactions or mortality following the initial and booster immunisation up to 28 days. Additionally, there were no clinical signs or reduction in body weight. The unimmunised mice that were given the ETEC H10407 strain challenge showed mortality within 24 h. In histological analysis, there was mild degeneration of myocytes in the heart, and gliosis and a necrotic area was observed in the brain of unimmunised mice, which may be related to an inadequate supply of oxygen after death. Both unimmunised and immunised mice showed signs of inflammation in their lungs, kidneys, and livers after the ETEC H10407 challenge, indicating that the infection is systemic. The pathological changes in the spleen were observed in both unimmunised and immunised mice after ETEC H10407 challenge, as expected, because the organ is in response to the infection. After a lethal challenge with ETEC H10407, the most remarkable alterations, including severe villi loss and necrosis, congestion with haemorrhage, and neutrophil infiltration, were observed in the intestine of unimmunised mice. Similar findings have been supported by a number of earlier investigations [42,49,50]. Notably, the immunised mice with MyChol™ did not show any mortality, diarrhoeal symptoms, and loss of body weight for 14 days after the ETEC challenge. No damage to or loss of villi was observed in the immunised mice intestine histopathological section, which indicates that the MyChol™ vaccine provides some protection by reducing the inflammation reaction and damage to the intestine. Compared to unimmunised mice, immunised mice showed a lower number of bacterial colonisation and fluid accumulation ratio in their intestines after 24 h of the ETEC challenge. These observations indicate that the immunised mice have elicited the anti-LT and anti-LT-B antibodies that hinder the LT and LT-B subunit of ETEC from binding to the GM1 ganglioside receptors on the epithelial cells of the intestine, preventing the endocytosis of LT into the cell. These observations are similar to those reported [51,52].

Anti-toxin IgG and IgA responses were associated with a decreased risk of diarrhoea after *V. cholerae* and ETEC challenge [46]. The immunogenicity data obtained in this study showed that the mice immunised with MyChol™, which contains VCUSM14P that expresses two copies of CTB, successfully triggered systemic IgG and IgA immune responses to CT, which in turn cross-reacted with the LT toxin produced by ETEC. Compared to anti-CT/CTB titres, anti-LT/LTB IgG titres were lower. This observed discrepancy could be explained by some specific antibodies made to CT/CTB that were non-reactive against LT/LTB, despite the fact that they are around 80% identical [16,44].

In this study, the immunised mice elicited six-fold anti-CTB IgG and two-fold anti-CTB IgA after the booster dose. Our results are consistent with those of mice that were given the vaccines Dukoral (10^9^ CFU), Shanchol (10^9^ CFU), a recombinant cholera toxin B subunit (rCTB) (16.7 g) [53], and Euvichol [54]. In contrast, the mice immunised with HaitiV elicited anti-CTB IgG (~30 ELISA units) and anti-CTB IgA (~300 ELISA units) on day 42 [55]. Distinctly, the mice immunised with Peru-15p CTB—a live-attenuated cholera vaccine—expressed high levels of CT-B (~30 fold) and recorded a high level of anti-CT-B titre with a geometric mean titre (GMT) of 3200 by day 42 [56].

In our study, the 12-fold anti-LT IgG and 4-fold IgA titres were elicited in immunised mice with MyChol™. Similar trends were observed in the mice immunized with different ETEC vaccines, such as MEFA, MecVax, CfaEB, and ZCR533-CFA/I + LThK63, which elicited 4.5 (log10) anti-LT IgG [57], 3.81 (log10) anti-LT IgG [58], 4-fold anti-LTB IgG titre [59], and 2.3 GMT in anti-LT serum IgG [60], respectively, with no IgA detected. Enteric infections tend to induce high titres of secreted mucosal IgA in the gut with little or no concomitant increase in serum IgA [61,62].

The absence of bactericidal antibodies in the serum samples from immunised mice shows that they do not develop antibodies against the LPS O antigen of the ETEC H10407 strain. The immunised mice withstood the ETEC challenge by producing anti-CT antibodies, which cross-neutralised the LT toxin. The research results show the cross-protection of MyChol^TM^ against the ETEC challenge in mice based on clinical symptoms and histological and immunological analyses.

## 5. Conclusions

The vaccine was found to be safe in mice without any adverse effects after immunisation. In the immunised mice, the vaccine triggered systemic IgG and IgA immune responses to the cholera toxin (CT), which in turn cross-reacted with the heat-labile (LT) toxin of the ETEC strain. The vaccine elicited neutralising antitoxin (anti-LT) and considerably minimised the fluid accumulation caused by the ETEC strain in the intestines of immunised mice. The vaccine cross-protected the mice from the ETEC challenge without causing any symptoms of diarrhoea and damage to the intestine, as evidenced in the histopathological results. This study demonstrates the safety, immunogenicity, and cross-protection of MyChol^TM^, a cold-chain-free, live-attenuated, oral cholera vaccine, against ETEC challenge in mice. The dual-use (cholera and ETEC) vaccine would provide simultaneous protection against *V. cholerae* O139 and ETEC infection for the bottom billion impoverished people, as well as for international travellers.

## Figures and Tables

**Table 1:** Histopathological findings of mice after ETEC challenge studies. Relative organ weight of BALB/c mice after immunisation and ETEC H10407 challenge studies.

Organs	Groups		
	Group A (Control Group)	Group B (Unimmunized Mice Challenged)	Group C (Immunized Mice Challenged)
**Brain**	Within normal limit	Gliosis and necrotic area are noted	Within normal limit
**Heart**	Within normal limit	Mild degeneration of myocytes with mild interstitial oedema	Within normal limit
**Lung**	Within normal limit	Extensive lymphocytic infiltration, congestion, and oedema of the interstitium with focal alveoli oedema and type II pneumocytes hyperplasia	Mild lymphocytic infiltration, congestion, and oedema of the interstitium with focal alveoli oedema and type II pneumocytes hyperplasia
**Spleen**	Within normal limit	Moderate focal parenchymal congestion with enlarged germinal centres, and focal white pulp disintegration and apoptosis	Mild focal parenchymal congestion and focal apoptosis
**Kidney**	Within normal limit	Mild degeneration of tubules Mild congestion Moderate inflammation of the interstitium	Mild degeneration of tubules Mild congestion Mild inflammation of the interstitium
**Liver**	Within normal limit	Mild degeneration of hepatocytes Severe sinusoidal congestion Mild Kupfer cell hyperplasia Moderate portal inflammation Moderate bile duct hyperplasia	Mild degeneration of hepatocytes Mild sinusoidal congestion Mild Kupfer cell hyperplasia Mild portal inflammation
**Pancreas**	Within normal limit	Within normal limit	Within normal limit
**Intestines**	Within normal limit	Severe necrosis, congestion and haemorrhage, and neutrophil infiltration	Within normal limit

## Data Availability

Not applicable.
